# Transaxillary endovascular aortic aneurysm repair using a reverse mounted Gore Excluder endograft for a patient with abdominal aortic aneurysm and severe iliofemoral occlusive disease

**DOI:** 10.1016/j.jvscit.2024.101706

**Published:** 2024-12-17

**Authors:** Andres V. Figueroa, Carla Scott, Jacqueline Babb, Antonio Solano, Natalia Coronel, Carlos H. Timaran, Mirza S. Baig

**Affiliations:** Division of Vascular and Endovascular Surgery, Department of Surgery, University of Texas Southwestern Medical Center, Dallas, TX

**Keywords:** Transaxillary artery approach, Endovascular aortic aneurysm repair, Peripheral artery disease, Adverse iliofemoral anatomy

## Abstract

Adverse iliofemoral anatomy represents a unique challenge for endovascular aortic aneurysm repair (EVAR). This report describes a transaxillary EVAR in a patient with severe iliofemoral occlusive disease and an infrarenal aortic aneurysm. A reversely mounted Gore Excluder graft was advanced and deployed in the infrarenal aorta using the left axillary artery. Lithoangioplasty and stenting were performed on bilateral iliofemoral anatomy. At the 1-year follow-up, the aneurysm sac revealed regression without endoleaks and the iliofemoral stents remained patent. The transaxillary approach may be a feasible access site for EVAR in patients with a high risk for open repair and prohibitive iliofemoral anatomy.

Endovascular aortic aneurysm repair (EVAR) has become the preferred treatment option for abdominal aortic aneurysms (AAAs).^1^, ^2^, ^3^, ^4^, ^5^ Hostile iliofemoral anatomy such as severe iliac tortuosity, stenotic iliac arteries, and calcified vessels may preclude eligibility for EVAR.^6^ Different adjunctive techniques, including open surgical conduits, endoconduits, and intravascular lithotripsy, have been used to improve vascular access during EVAR. Despite associated disadvantages for each technique, obtaining adequate iliofemoral access is paramount to advancing the endograft.^7^, ^8^, ^9^, ^10^, ^11^, ^12^ Therefore, the presence of severe iliofemoral disease is usually considered prohibitive to performing EVAR. The upper extremity (UE) approach to delivering endovascular devices has been described in selected cases to perform EVAR.^13^ We detail the technique for a transaxillary percutaneous approach to perform EVAR of an infrarenal aortic aneurysm in a patient with severe bilateral occlusive disease using a modified Gore Excluder endograft delivery system.

## Case Report

**Fig 1:**
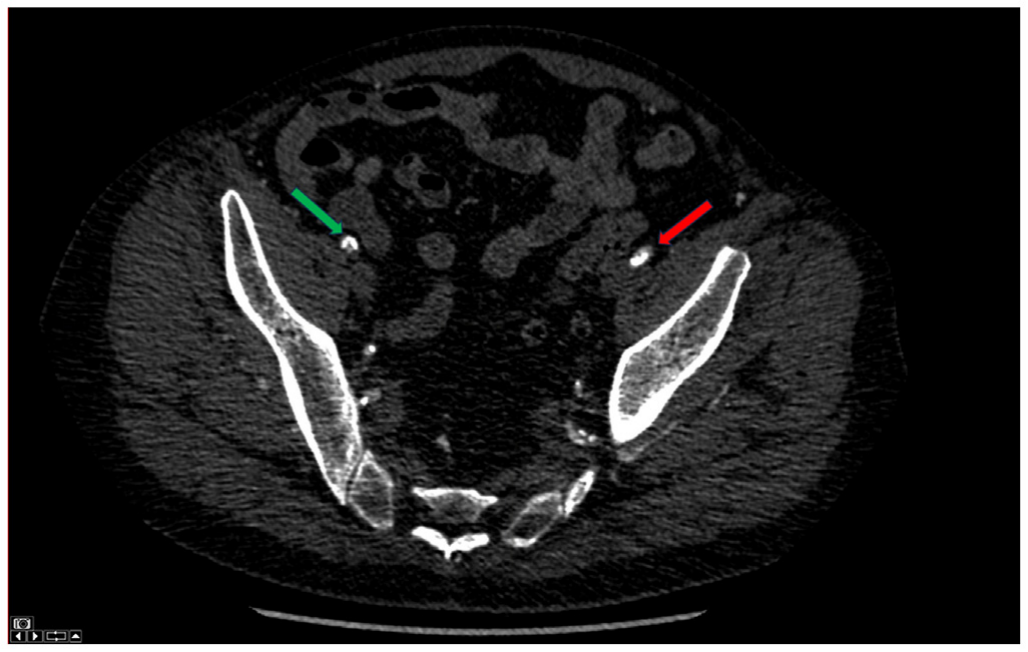
Computed tomographic angiography (CTA) reveals severe peripheral arterial disease of the right external iliac artery (EIA) (*green arrow*) and the left EIA (*red arrow*).

**Fig 2:**
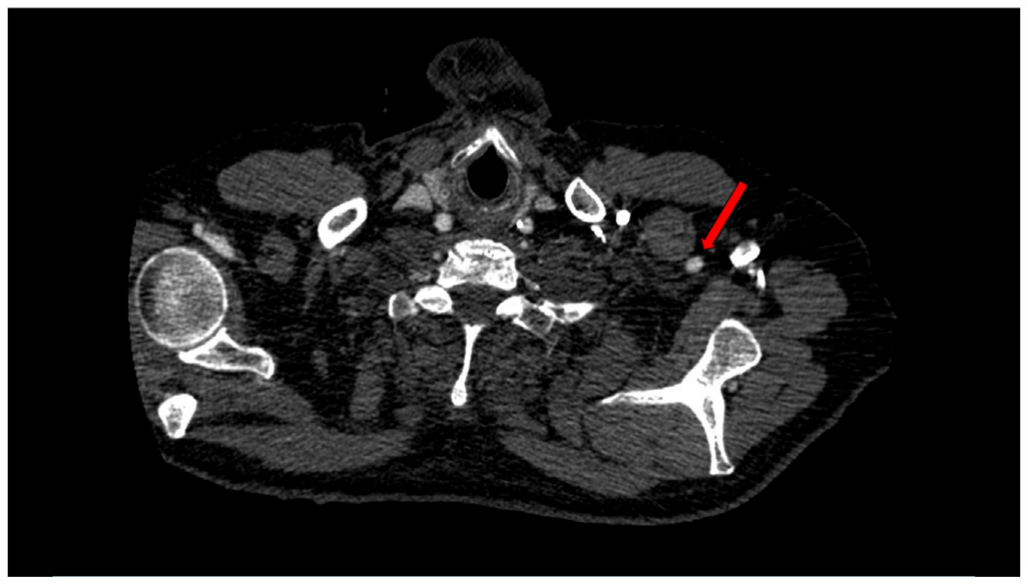
Computed tomographic angiography (CTA) reveals a left axillary artery suitable to advance the endovascular stent draft during endovascular aortic aneurysm repair (EVAR).

A 75-year-old man with a medical history of hypertension, hyperlipidemia, active smoking, and peripheral artery disease with symptoms of disabling claudication presented with an asymptomatic 5.7-cm infrarenal AAA with a 19-mm diameter and 30-mm-long neck. The patient had a calcified occlusion of the right external iliac artery (EIA), heavily calcified right common femoral artery (CFA) stenosis, and calcified stenosis of the left common iliac origin and the length of the left EIA. The right internal iliac artery was patent and the left internal iliac artery had a stenosis at the origin. Given severe bilateral iliofemoral disease (Fig 1), open repair with aortobifemoral bypass vs iliofemoral endarterectomies with EVAR were presented to the patient as options. However, the patient refused any open surgical component for his operation. The patient was subsequently referred to us to evaluate for a totally percutaneous solution. Chest computed tomographic angiography (CTA) was performed to evaluate alternative treatment approaches revealing a left axillary artery measuring 7 to 8 mm (Fig 2). Given the favorable UE access, we discussed with the patient the alternative of transaxillary delivery of an excluder endograft with modification of the delivery system and recanalization of the iliofemoral anatomy. After explaining the risks and benefits of this approach, the patient gave informed consent for the surgery.

In a hybrid operating room, bilateral groins and the left arm were prepped under general anesthesia. The left axillary artery was accessed under the pectoralis minor muscle to carefully avoid the brachial plexus root under ultrasound guidance, followed by the placement of 2 Perclose devices.^14^ The axillary access was upsized with an 18Fr x 65 cm Gore DrySeal sheath (W. L. Gore & Associates, Flagstaff, AZ), which was advanced to the infrarenal aorta. The left EIA demonstrated >90% stenosis with heavily calcified plaque requiring lithoangioplasty of the left EIA, common iliac artery (CIA), and CFA with a 6.5 mm × 60.0 mm Shockwave balloon (Shockwave Medical Inc, Santa Clara, CA). Then, covered self-expandable stents 8 mm × 10 cm and 8 mm × 7.5 cm (Viabahn, W. L. Gore & Associates) were deployed from the left CFA bifurcation to the origin of the EIA, and a covered balloon-expandable stent 8 × 39 mm (Viabahn VBX, W. L. Gore & Associates) was deployed in the left CIA. A bare metal stent 8 × 29 mm (Onmilink, Abbott, Chicago, IL) was deployed from the common to the external iliac covered stents to maintain perfusion to the internal iliac artery.

**Fig 3:**
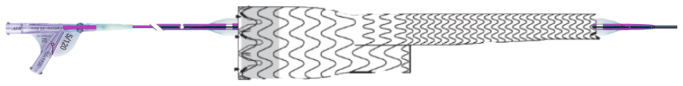
Reverse mounted Gore Excluder graft on an Armada balloon catheter to allow transaxillary delivery in the same orientation as a transfemoral delivery.

Next, a 23.0 mm × 14.5 mm × 12.0 cm Gore Excluder graft (W. L. Gore & Associates) was dismounted from its delivery system without deployment, and reverse mounted on a 4 × 150 mm Armada balloon catheter to allow transaxillary delivery in the same orientation as a transfemoral delivery (Fig 3). The graft was advanced over an Amplatz wire whose distal end was in the left superficial femoral artery. The graft was positioned at the infrarenal neck. The ipsilateral limb was initially deployed into the previously deployed left CIA stent, followed by the graft main body deployment and contralateral cannulation. Occlusion at the origin of the right EIA was identified, requiring micropuncture access of the right femoral bifurcation to retrograde across the right EIA with a .018″ Glidewire. The wire was snared from the axillary access, obtaining through-and-through access and then wire access into the right SFA was established from the axillary access. Intravascular Lithotripsy of the right CFA and EIA was then performed with a 6.5 mm × 60 mm Shockwave balloon (Shockwave Medical Inc). An 8 mm × 15 cm Viabahn stent was deployed from the CFA bifurcation to the origin of the EIA, and an 8 × 79 mm VBX stent in the CIA, followed by an 11 × 59 mm VBX stent from the CIA to the contralateral gate of the excluder. A bare metal stent 8 mm × 60 mm (EverFlex, Medtronic, Minneapolis, MN) was deployed between the right CIA and EIA to maintain perfusion to the internal iliac artery.

**Fig 4:**
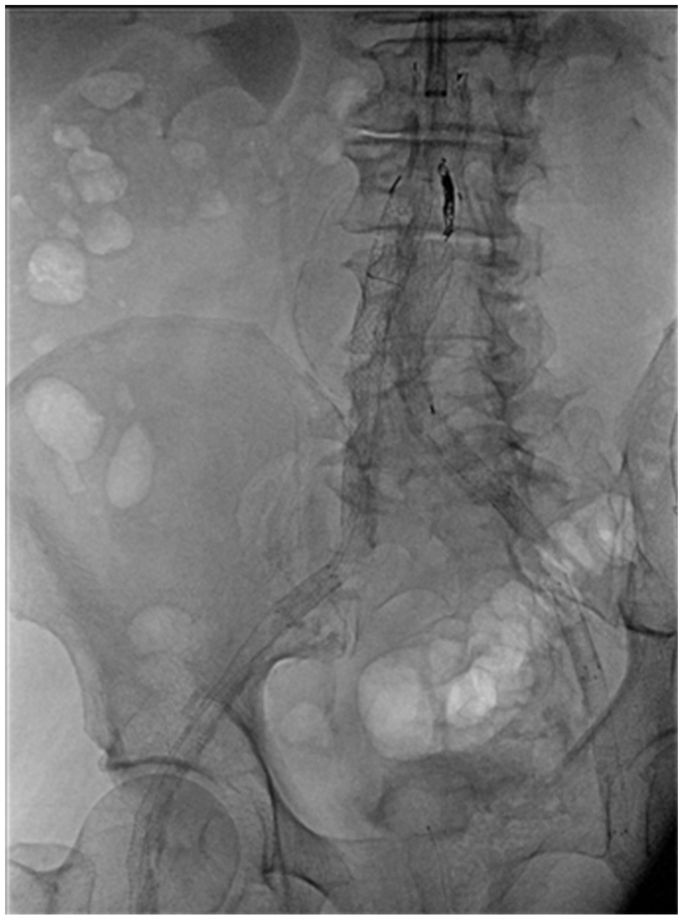
Plain radiograph revealing final graft configuration of the Gore Excluder Endograft to exclude the abdominal aortic aneurysm (AAA).

**Fig 5:**
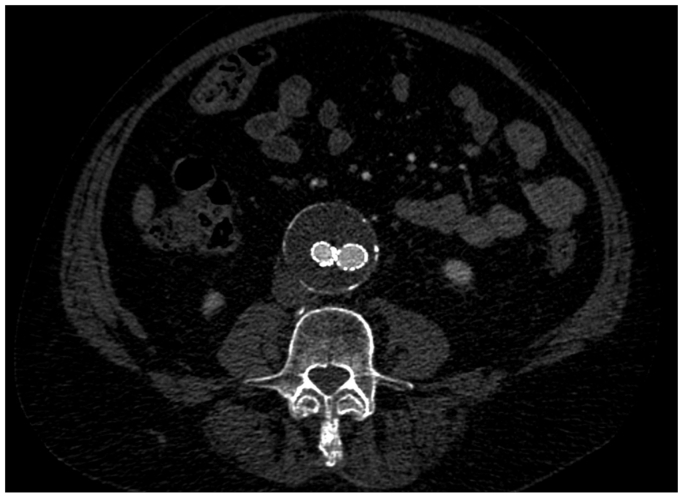
Computed tomographic angiography (CTA) reveals an excluded abdominal aortic aneurysm (AAA) without endoleaks.

An UE angiogram at the end of the procedure revealed no significant extravasation, and the radial artery was strongly palpable at the left wrist. The patient was discharged home on postoperative day 2 without access site complications. Completion angiogram demonstrated aneurysm exclusion with patent iliofemoral arteries and no evidence of endoleak; the final graft configuration is demonstrated on a plain radiograph (Fig 4). Bilateral posterior tibial arteries were palpable. At the 1-year follow-up visit, the patient reported resolution of his claudication symptoms, and CTA revealed aneurysmal sac size regression, no endoleaks, and patent bilateral profunda and superficial femoral arteries (Fig 5). The patient consented to publish the details of his case.

## Discussion

Our case report details the technical aspects of performing a transaxillary approach to deliver an endovascular stent graft during EVAR in a patient refusing any open surgical component for his aneurysm repair. The introduction of endovascular surgery for AAA repair has surpassed open surgery as the preferred treatment option. Despite being broadly used in the past, previous reports have revealed a higher mortality and morbidity rate with open surgery compared to the endovascular approach.^15^, ^16^, ^17^, ^18^ The preferred access site for EVAR is the CFA, given the relative ease of access, the size to accommodate the endograft, and compression support against the femoral head.^19^ Although severe iliofemoral disease may be considered a total contraindication for EVAR, a careful evaluation of alternative access vessels focusing on vessel diameter, the presence of calcification, and tortuosity may overcome this limitation.^20^

Axillary access may be an alternative access site for EVAR, given its often suitable diameter, short working distance, and relative freedom from calcification and atherosclerotic disease.^21^ In our case, after CTA evaluation of vessel anatomy, the left axillary artery measured 7 to 8 mm, which allows delivery of low-profile devices (ie, 16F or 18F devices). One of the most devastating complications of UE access is cerebrovascular events, which have been previously reported in ≤10.7% of cases.^22^ Notably, the introduction of low-profile devices not only allows the use of smaller vessels, but also limits aortic arch manipulation, thereby decreasing cerebrovascular and embolic complications.^13^^,^^23^ Additionally, in patients with UE access, the left axillary artery is preferred because of limited passage of devices through the aortic arch and the reduced risk of potential cerebral embolization compared to the right UE or bilateral UE access.^22^^,^^23^

Previous reports have described the suitability of performing thoracic EVAR using transapical, transaxillary, or transcarotid access.^23^, ^24^, ^25^ More recently, Bertoglio et al^13^ reported their experience using transaxillary access for complex EVAR, highlighting the need for surgical conduit creation to prevent injury of the access vessel wall and the need for patient-specific company-manufactured devices, which can be loaded in reverse orientation. In our case, the endovascular stent graft must be dismounted from the stock delivery system and reverse mounted to allow transaxillary delivery in the same orientation as a transfemoral delivery. An Armada balloon catheter (Abbott) was placed through the delivery catheter tract of the excluder graft, followed by hand inflation of the balloon on both sides to create a shoulder to prevent the dismounting of the graft from the balloon catheter during delivery. Finally, the two deployment lines were lengthened with silk sutures for the main deployment line and a different color suture for the ipsilateral limb deployment line. Notably, no surgical conduits were used in our case, highlighting the feasibility of a total percutaneous transaxillary approach for EVAR.

Our case detailed an alternative access site for EVAR in patients with a high risk for open repair or prohibitive iliofemoral anatomy. However, some limitations should be acknowledged. First, even with the introduction of low-profile devices, large access sites are required to advance the endograft, which may not be suitable in all the axillary arteries. Second, the rates of cerebrovascular complications using UE approach are not negligible and warrant careful consideration before using this approach. Third, the endovascular coverage detailed in this report may increase the total cost of the procedure. Finally, the requirement to dismount the endograft represents an off-label use of the device, which may increase the risk of intraprocedural complications.

## Conclusions

Total percutaneous transaxillary delivery of a remounted Gore Excluder endoprosthesis is a feasible technique for the repair of AAA in patients with high morbidity and mortality risk for open repair and no viable iliofemoral access for endovascular repair.

## Funding

None.

## Disclosures

M.S.B. and C.H.T. have been consultants for and received research support from Cook Medical Inc, W. L. Gore & Associates, Inc., and Phillips Healthcare.
